# Non-AUG Translation Initiation Generates Peroxisomal Isoforms of 6-Phosphogluconate Dehydrogenase in Fungi

**DOI:** 10.3389/fcell.2020.00251

**Published:** 2020-05-05

**Authors:** Marco Kremp, Elena Bittner, Domenica Martorana, Alexander Klingenberger, Thorsten Stehlik, Michael Bölker, Johannes Freitag

**Affiliations:** ^1^Department of Biology, Philipps-University Marburg, Marburg, Germany; ^2^LOEWE Center for Synthetic Microbiology, Marburg, Germany

**Keywords:** dual targeting, pentose-phosphate pathway, non-AUG translation, redox-shuttle, peroxisome

## Abstract

Proteins destined for transport to specific organelles usually contain targeting information, which are embedded in their sequence. Many enzymes are required in more than one cellular compartment and different molecular mechanisms are used to achieve dual localization. Here we report a cryptic type 2 peroxisomal targeting signal encoded in the 5′ untranslated region of fungal genes coding for 6-phosphogluconate dehydrogenase (PGD), a key enzyme of the oxidative pentose phosphate pathway. The conservation of the cryptic PTS2 motif suggests a biological function. We observed that translation from a non-AUG start codon generates an N-terminally extended peroxisomal isoform of *Ustilago maydis* PGD. Non-canonical initiation occurred at the sequence AGG AUU, consisting of two near-cognate start codons in tandem. Taken together, our data reveal non-AUG translation initiation as an additional mechanism to achieve the dual localization of a protein required both in the cytosol and the peroxisomes.

## Introduction

Organelles are specialized reaction chambers of eukaryotic cells. Each type of organelle harbors a unique combination of enzymes to catalyze specific metabolic pathways such as aerobic ATP generation in the mitochondria or β-oxidation of long-chain fatty acids inside peroxisomes (Lodish et al., 2000). A number of enzymatic activities is crucial inside more than one organelle or inside of an organelle and the cytosol. Several molecular mechanisms that enable the synthesis of protein isoforms with different cellular destinations from a single gene have been discovered. These include alternative transcriptional start sites, alternative splicing, or competing targeting signals (Carrie et al., 2009; Yogev and Pines, 2011; Ast et al., 2013). A more recently described mechanism to generate dually targeted protein variants is programmed translational read-through of stop codons. This mechanism is widely used for the formation of C-terminally extended enzymes containing peroxisomal targeting signals (PTS1) (Freitag et al., 2012; Schueren et al., 2014; Stiebler et al., 2014; Schueren and Thoms, 2016).

Peroxisomes are ubiquitous organelles with a major function in the β-oxidation of fatty acids (Poirier et al., 2006; Smith and Aitchison, 2013; Wanders et al., 2016). Most peroxisomal proteins are targeted to this organelle by either of two signal sequences residing at the very C-terminus (PTS1) or within the N-terminus (PTS2) (Baker et al., 2016). PTS2 motifs are characterized by the consensus motif (R/K)-(L/V/I)-xxxxx-(H/Q)-(L/A) and recognized by the soluble import receptor Pex7 (Lazarow, 2006).

The enzymes of the pentose phosphate pathway, including 6-phosphogluconate dehydrogenase (EC: 1.1.1.44), have already been shown to purify with mammalian peroxisomes 30 years ago, but this could not be confirmed by recent proteomics experiments (Antonenkov, 1989; Yifrach et al., 2018). In plants, peroxisomal 6-phosphogluconate dehydrogenase may act as part of an NADPH recycling system, and a peroxisomal variant of this enzyme has been shown to be involved in gametophytic interaction (Corpas et al., 1998; Hölscher et al., 2016).

A closer examination of genes for putative 6-phosphogluconate dehydrogenases in *Ustilago maydis* and related fungi leads to the identification of DNA sequences encoding PTS2-like motifs and which are embedded in the 5′ UTRs. We show that a peroxisomal isoform of *U. maydis* 6-phosphogluconate dehydrogenase (Pgd1) containing a functional PTS2 signal is translated *via* initiation at a non-AUG start codon.

## Results and Discussion

**FIGURE 1:**
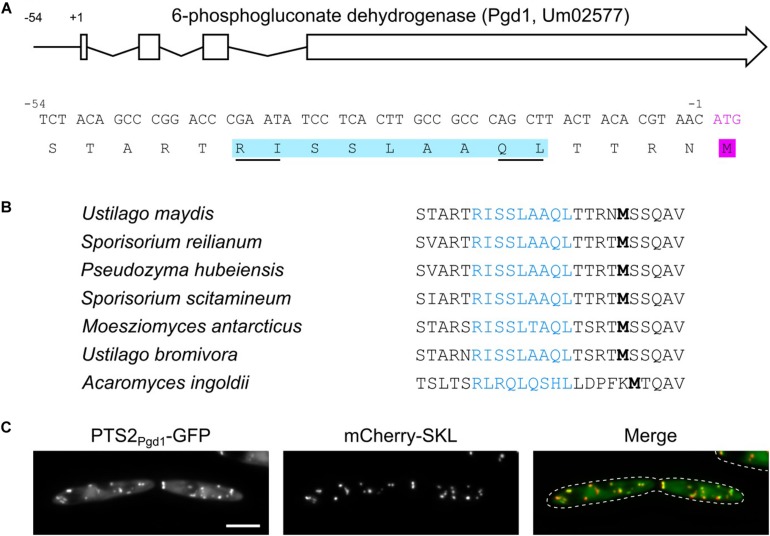
A functional PTS2 in the 5′ untranslated region (UTR) of *pgd1.* **(A)** Architecture of the *U. maydis pgd1* gene and sequence of the encoded PTS2 motif in the 5′ UTR. **(B)** Phylogenetic conservation of the PTS2 encoded in the 5′ UTR of *pgd1* orthologs in related fungi. Note that the amino acids can be variant but still fit into the PTS2 consensus (R/K)-(L/V/I)-xxxxx-(H/Q)-(L/A). **(C)** Representative fluorescence microscopy pictures of strain MB215 PTS2*_*Pgd*__1_*-GFP mCherry-SKL. Green channel (PTS2*_*Pgd*__1_*-GFP), red channel (mCherry-SKL), and merged channels are shown. The scale bar represents 5 μm.

We have previously identified a network of sugar-metabolizing enzymes, which is dually targeted to the cytosol and peroxisomes *via* post-transcriptional mechanisms. The common theme of these mechanism is the generation of peroxisomal targeting signals by translational read-through or differential splicing (Freitag et al., 2012, 2018; Stiebler et al., 2014). An investigation of the *U. maydis* gene *umag_02577* (GeneID: 23563291) coding for 6-phosphogluconate dehydrogenase (Pgd1) revealed that its 5′ UTR codes for the amino acid stretch RISSLAAQL, matching the PTS2 consensus (Lazarow, 2006). The coding sequence for this putative targeting signal starts 39 nucleotides upstream of the annotated start codon (Figure 1A). Remarkably, no in-frame start codon was detected upstream of the putative PTS2 in the *pgd1* 5′ UTR. Moreover, published *U. maydis* RNA-Seq data (Lanver et al., 2018) did not indicate alternatively spliced *pgd1* transcripts containing an AUG start codon in frame with the sequence coding for the PTS2 (Supplementary Figure S1). A phylogenetic analysis showed that the putative PTS2 motifs in the 5′ UTR of *pgd1* are conserved in related fungi, suggesting functionality (Figure 1B). We inserted the PTS2-encoding sequence into a green fluorescent protein (GFP) reporter construct behind the canonical ATG start codon. The resulting fusion protein PTS2*_*Pgd*__1_*-GFP accumulated in punctate structures, co-localizing with the peroxisomal marker mCherry-SKL (Figure 1C). Thus, the 5′ UTR of *pgd1* contains a functional PTS2.

**FIGURE 2:**
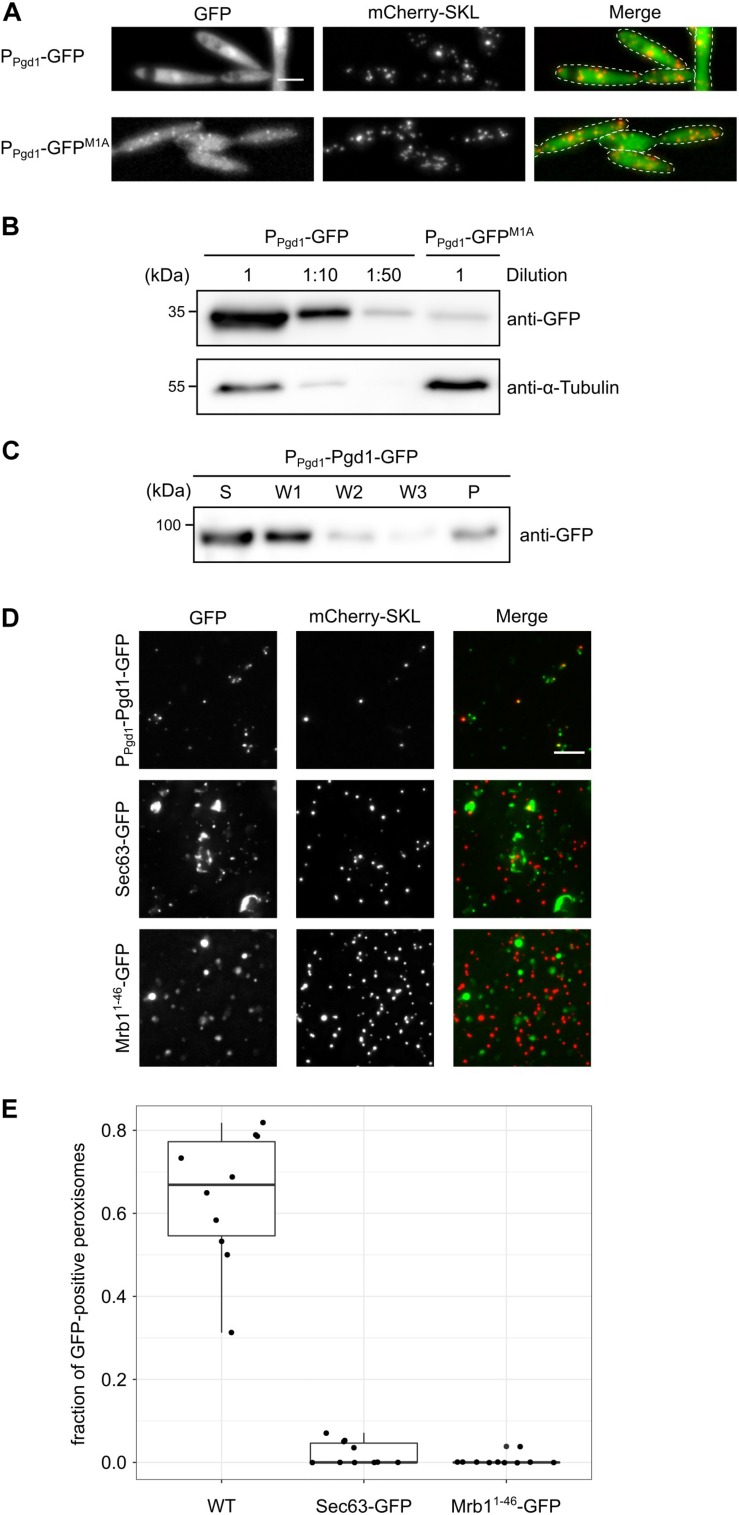
An extended peroxisomal isoform of Pgd1 is generated *via* non-AUG translation initiation. **(A)** Fluorescence microscopy pictures of strains containing mCherry-SKL together with P*_*pgd*__1_*-GFP and P*_*pgd*__1_*-GFP^M1A^, respectively. Green channel, red channel (mCherry-SKL), and merged pictures are shown. The scale bar represents 5 μm. **(B)** Western blot showing the GFP and α-tubulin levels in whole-cell extracts derived from strains analyzed in **(A)**. **(C)** The subcellular localization of Pgd1-GFP was analyzed by differential centrifugation of the post-nuclear supernatants. Supernatant, wash, and pellet fractions were analyzed by sodium dodecyl sulfate-polyacrylamide gel electrophoresis and Western blot. **(D)** Fluorescence microscopic images of organelles prepared from the indicated strains. The scale bar represents 5 μm. **(E)** Quantification of mCherry-SKL-positive foci also containing GFP signal.

The absence of a canonical start codon upstream of the encoded PTS2 suggests the initiation of translation at a non-AUG start codon. To test this hypothesis, we generated chimeric constructs consisting of 1,000 nucleotides *pgd1* upstream sequence fused to the GFP open reading frame (ORF). Two constructs either with or without the canonical start codon of the GFP-ORF (P*_*pgd*__1_*-GFP and P*_*pgd*__1_*-GFP^M1A^, respectively) were transformed into *U. maydis*. For P*_*pgd*__1_*-GFP, a strong cytosolic GFP fluorescence was observed (Figure 2A). This signal likely reflects the translation initiation at the canonical start codon of the GFP-ORF. The mutation of the canonical start codon in P*_*pgd*__1_*-GFP^M1A^ resulted in the detection of GFP inside of peroxisomes (Figure 2A). We also detected a corresponding GFP signal by Western blot analysis (Figure 2B). The quantification of the GFP signal from P*_*pgd*__1_*-GFP and P*_*pgd*__1_*-GFP^M1A^ revealed that non-canonical initiation occurs at a rate of approximately 2%. No obvious migration difference between cytosolic and peroxisomal GFP isoforms was detected on sodium dodecyl sulfate-polyacrylamide gel electrophoresis (SDS-PAGE) (Figure 2B). In several organisms, the PTS2 signals are cleaved off after import (Kurochkin et al., 2007; Schuhmann et al., 2008). However, no homolog of the processing protease was identified in the genome of *U. maydis.* To analyze potential cleavage upon peroxisomal import, we examined the migration of the reporter protein PTS2_*Pot*__2_-GFP in wild-type and Δ*pex7* mutants (Supplementary Figure S2A). Pot2 (Umag_01090) encodes a conserved peroxisomal enzyme involved in fatty acid oxidation. No difference in mobility was observed on SDS-PAGE (Supplementary Figure S2B). As a control, we used GFP-SKL, which to our surprise migrated with lower mobility, although the protein has a lower molecular weight. Thus, the migration of the analyzed GFP fusion proteins is not only determined by their mass, and no conclusions about processing of the PTS2 can be drawn.

Next, we examined the subcellular distribution of full-length Pgd1. We generated a strain expressing a C-terminally GFP-tagged variant of Pgd1 under control of the endogenous promoter (Pgd1-GFP). A microscopic analysis of the resulting strain revealed a uniform distribution of Pgd1-GFP, indicating a predominantly cytosolic localization (Supplementary Figure S3A). Crude organelles were isolated and subjected to differential centrifugation to remove cytosolic Pgd1-GFP. We detected the fusion protein in the pellet fraction, suggesting that it also resides inside of an organelle (Figure 2C). In addition, we analyzed organelle preparations by fluorescence microscopy and detected GFP signal in most mCherry-positive foci (Figures 2D,E). Green foci which do not contain mCherry-SKL were also observed. These foci could represent aggregates of Pgd1-GFP or peroxisomes, which have lost the mCherry-SKL signal. As a control, we prepared organelles from a strain expressing either the endoplasmic reticulum membrane protein Sec63-GFP or the mitochondrial marker protein Mrb1^1–48^-GFP (Supplementary Figure S3B; Rothblatt et al., 1989; Bortfeld et al., 2004). Less GFP foci overlapped with mCherry (Figures 2D,E). Taken together, these data indicate that unconventional translation initiation in the 5′ UTR of *pgd1* triggers the generation of a peroxisomal isoform.

**FIGURE 3:**
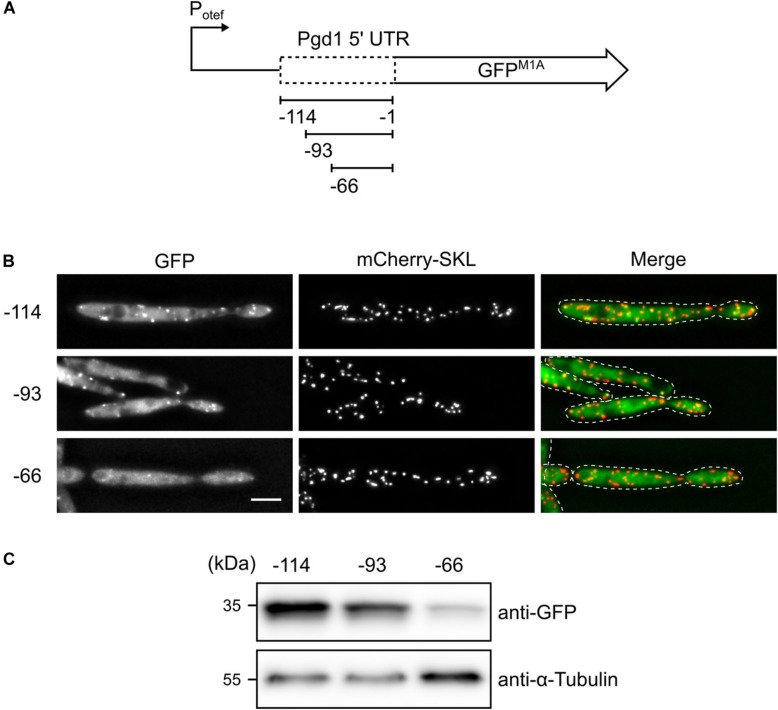
Truncation analysis to identify the sequences required for non-AUG translation initiation. **(A)** Schematic representation of the constructs tested. **(B)** Fluorescent images of strains, which express mCherry-SKL and contain the indicated *pgd1* upstream sequences between the constitutive *otef*-promoter and the GFP^M1A^-ORF. Green channel, red channel, and merged pictures are shown. The scale bar represents 5 μm. **(C)** Western blot showing the protein levels of GFP and α-tubulin in whole-cell extracts derived from the strains analyzed in **(B)**.

To determine the site for translation initiation, a series of truncations of the *pgd1* 5′ UTR fused to GFP^M1A^ was constructed and expressed under control of the constitutive *otef*-promoter (Spellig et al., 1996; Figure 3A). Strains containing the reporter constructs were analyzed by fluorescence microscopy and Western blot. We detected both cytosolic and peroxisomal GFP fluorescence in all strains. In the strain containing only 66 base pairs (bp) of the *pgd1* 5′ UTR mostly, background fluorescence was observed, and the peroxisomal GFP signal was strongly reduced (Figure 3B). Background fluorescence is regularly observed in *U. maydis* cells if fusion proteins with a relatively low expression level are investigated. The 66-construct, however, still codes for PTS2, indicating that the unconventional translation initiation upstream of the PTS2 was affected. This idea was supported by Western blot analysis showing that GFP expression was reduced if only 66 bp of the 5′ UTR was present (Figure 3C). Hence, the mRNA sequence between position −66 and −93 appears to be involved in non-canonical translation initiation.

**FIGURE 4:**
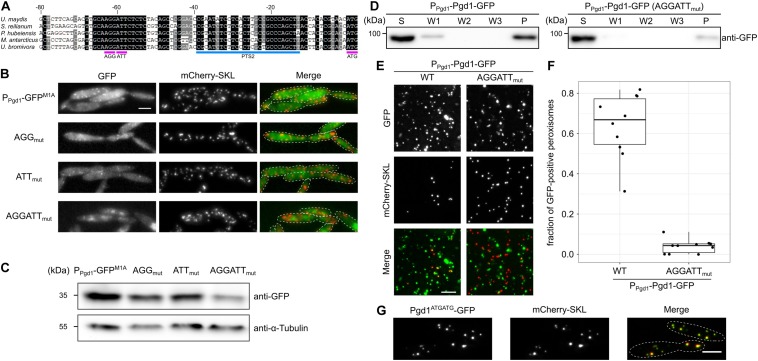
Two non-canonical start codons in tandem mediate non-AUG translation initiation and are involved in peroxisomal targeting. **(A)** Alignment of 5′ UTR sequences of 6-phosphogluconate dehydrogenase encoding the genes of several fungi. Note that the region from –68 to –53 containing the near-cognate start codons AGG and ATT is highly conserved. **(B)** Fluorescence microscopy images of strains which express mCherry-SKL and contain the indicated mutations in the 1,000-nucleotide *pgd1* upstream sequence fused to the GFP^M1A^-ORF. Green channel, red channel, and merged pictures are shown. The scale bar represents 5 μm. **(C)** Western blot showing the GFP and α-tubulin levels of the indicated strains. **(D)** The subcellular localization of the indicated strains was analyzed by differential centrifugation of post-nuclear supernatants. Supernatant, wash, and pellet fractions were analyzed by sodium dodecyl sulfate-polyacrylamide gel electrophoresis and Western blot. **(E)** Fluorescence microscopic images of the organelles prepared from the indicated strains. The scale bar represents 5 μm. **(F)** Quantification of mCherry-SKL-positive foci also containing Pgd1-GFP. **(G)** The two near-cognate start codons AGG and ATT were substituted with two ATG start codons. The subcellular localization of the resulting fusion protein (Pgd1^ATGATG^-GFP) was determined by fluorescence microscopy. The scale bar represents 5 μm.

It has been shown that non-AUG translation initiation often occurs at near-cognate start codons that differ from AUG by a single base change (de Arce et al., 2017; Kearse and Wilusz, 2017). No such codons are located between nucleotides −93 and −66 of the *pgd1* 5′ UTR. However, a pair of the near-cognate start codons AGG AUU stretches from −63 to −58 and is in frame with the canonical start codon of *pgd1*. Both codons reside inside of a phylogenetically conserved region within the 5′ UTR of *pgd1* mRNA (Figure 4A). To test whether these codons serve as translational initiation sites, they were substituted with the alanine codon GCA, either alone or in tandem. Constructs with single substitutions still led to the detection of peroxisomal GFP, although with weaker intensity compared to the wild-type sequence. Mutation of both codons abolished the detection of a peroxisomal GFP fluorescence (Figure 4B). The Western blot experiments were in line with our microscopy data (Figure 4C). However, a weak GFP signal was still visible in the double mutant, indicating that, even in the absence of both near-cognate start codons, translation initiation occurs at a very low level (Figure 4C). Next, we generated a full-length C-terminally GFP-tagged version of Pgd1 expressed under control of the endogenous promoter with mutations in both non-canonical start codons (AGG ATT substituted with GCA). The differential centrifugation of post-nuclear supernatants showed a reduced amount of Pgd1-GFP in the organelle pellet compared to the amount of Pgd1-GFP without the respective mutations (Figure 4D). A microscopic analysis of crude organelle pellets confirmed this data. The mutation of both near-cognate start codons to GCA considerably reduced the number of mCherry-SKL foci containing Pgd1-GFP (Figures 4E,F). Finally, we generated a construct in which both near-cognate start codons in the 5′ UTR of Pgd1-GFP were substituted with ATG. As expected, Pgd1^ATGATG^-GFP localized in peroxisomes together with mCherry-SKL (Figure 4G). Taken together, these data indicate that both near-cognate start codons play a key role in the generation of peroxisomal Pgd1.

Here we have shown that, in *U. maydis*, non-AUG translation initiation is used for dual targeting of Pgd1 to peroxisomes and the cytosol. A phylogenetic analysis suggests that this occurs also in other fungi. Non-AUG translation initiation has recently emerged as a pervasive modulator of proteomes in diverse organisms (Kearse and Wilusz, 2017). Ribosome profiling especially has led to the identification of alternative translation initiation events in yeast and human cells (Ingolia et al., 2009, 2011). Non-canonical initiation was found to be regulated during yeast meiosis, under certain stress conditions and in different tissues of the mouse brain (Brar et al., 2012; Andreev et al., 2015; Zhang et al., 2015; Brar, 2016; Sapkota et al., 2019). Also, the evolutionary conservation of N-terminal extensions was described (Ivanov et al., 2011). Recently, it was shown that many yeast genes encode putative mitochondrial targeting signals in their 5′ UTR (Monteuuis et al., 2019). A systematic analysis of proteome data for yet undetected peptides encoded upstream of canonical translational start sites (Choi et al., 2020) in combination with mining of ribosome profiling data (Dunn et al., 2013; Schueren and Thoms, 2016) will reveal the full impact of non-AUG translation initiation for the generation of novel peroxisomal isoforms.

Interestingly, dual targeting of PGD seems to be evolutionarily conserved, but the underlying mechanism responsible for peroxisomal sorting is variable. In plants, different genes encode the peroxisomal and plastid isoforms of PGD (Hölscher et al., 2016). In *Candida albicans* and related ascomycetes, peroxisomal PGD arises from alternative splicing (Strijbis et al., 2012). The notable variability of mechanisms suggests that there might not be a conserved regulatory principle, which adapts the rate of synthesis of peroxisomal isoforms of PGD to different environmental conditions or developmental stages. It appears to be sufficient for cells that a fraction of PGD activity is present inside the peroxisomes to enable the production of NADP or NADPH. Similarly, we observed that different post-transcriptional processes, including alternative splicing and programmed read-through of stop codons, trigger the formation of peroxisomal isoforms of phosphoglycerate kinase and glyceraldehyde-3-phosphate dehydrogenase in fungi (Freitag et al., 2012). The mechanisms for dual targeting of peroxisomal proteins are likely to evolve rapidly and seem to be interchangeable. A recent report even proposes that stop codon read-through in general is non-adaptive (Li and Zhang, 2019). However, ribosomal read-through rates have been shown to rapidly change in response to cellular oxygen levels (Andreev et al., 2015). This may occur *via* the hydroxylation of a proline residue in the ribosomal decoding center that affects termination efficiency (Loenarz et al., 2014; Singleton et al., 2014). How the diversity of mechanisms for peroxisomal targeting of enzymes is adapted to peroxisomal metabolism still requires experimental evidence.

## Materials and Methods

### Strains, Growth Conditions, and Transformation

An *Escherichia coli* strain TOP10 (Invitrogen) was used for cloning procedures and amplification of plasmid DNA. The *U. maydis* strain MB215 mCherry-SKL (Hewald et al., 2005; Freitag et al., 2014) served as the wild-type strain for all of the experiments performed. The *U. maydis* strains generated and used during this study are listed in Supplementary Table S1. The *U. maydis* strains were grown at 28°C in YEPS-light medium (Tsukuda et al., 1988) or in YNB-medium (Difco) supplemented with 2% glucose and 0.5% ammonium sulfate at pH 5.8. The transformation of *E. coli* and *U. maydis* was done as previously described (Schulz et al., 1990; Hanahan et al., 1991). The constructs were integrated into the *ip*-locus (Broomfield and Hargreaves, 1992) of *U. maydis* cells.

### Molecular Cloning and Nucleic Acid Procedures

Either standard protocols were followed for molecular cloning or Gibson assembly was used for the generation of plasmids (Sambrook et al., 1989; Gibson et al., 2009). Maps of the generated plasmids are available on request. All primers used in this study are listed in Supplementary Table S2. Genomic DNA from *U. maydis* cells was prepared as described (Hoffman and Winston, 1987). Integration into the *ip*-locus was verified by Southern blot analysis (Sambrook et al., 1989).

### Preparation of Proteins and Western Blot Analysis

Whole-cell extracts of proteins were either prepared as described before (Kushnirov, 2000) or by glass bead-assisted lysis. Briefly, 30 ml of *U. maydis* cell culture with an optical density (OD_600_) of 1 was pelleted, washed once with water, and resuspended in 200 μl Tris–buffered saline containing 0.1% (v/v) Triton X-100 and 0.1% protease inhibitor cocktail for use with fungal and yeast extracts (Sigma-Aldrich). Then, 0.1 g of glass beads was added. The suspensions were deep-frozen at −80°C for at least 2 h, thawed at 4°C, and shredded at 4°C on a VXR basic Vibrax for 30 min. The suspensions were pelleted for 10 min at 13,000 rpm at 4°C, and the protein concentration in the supernatant was determined by Bradford assays (Bradford, 1976). Western blot analysis was performed as described (Stiebler et al., 2014). Antibodies against mCherry (Biovision; 5993), GFP (Torrey Pines Biolabs; TP401), and alpha-tubulin (Calbiochem; CP06) were used, respectively. Secondary antibodies against rabbit or mouse conjugated with horseradish peroxidase (Santa Cruz; sc-2357 and sc-516102) were used. Detection was performed with Supersignal West Femto or Supersignal West Pico Chemiluminescent Substrates (Thermo Fisher Scientific) and a Chemo Cam Imager (INTAS Science Imaging). The signals were analyzed using ImageJ (Schneider et al., 2012). Western blots were quantified with the ImageJ plugin GelAnalyzer. The generated gel profile plots were analyzed using the wand tool.

### Preparation of Crude Organelles From *Ustilago maydis*

A method published by Cramer et al. (2015) was adapted to *U. maydis* (steps 1–16; Cramer et al., 2015). Logarithmically growing *U. maydis* cells (400 ml) were pelleted, washed with water, and resuspended in buffer for spheroplasts containing 1 mg/ml novozyme 234 (Novo Nordisc). After spheroplasting, the protocol of Cramer et al. (2015) was followed. The post-nuclear supernatant (PNS) was diluted to an OD_600_ of 1, and aliquots were frozen at −80°C. For subcellular fractionation analysis, 1 ml of PNS was centrifuged at 13,000 rpm at 4°C for 5 min. The pellets were resuspended in lysis buffer (5 mM MES, 0.5 mM EDTA, 1 mM KCl, 0.6 M sorbitol, 1 mM 4-aminobenzamidine-dihydrochloride, 1 μg/ml aprotinin, 1 μg/ml leupeptin, 1 mM phenylmethylsulfonyl fluoride, 10 μg/ml N-tosyl-L-phenylalanine chloromethyl ketone, and 1 μg/ml pepstatin) and washed with lysis buffer as indicated in Figure 2.

### Microscopy

*Ustilago maydis* cells from logarithmically growing cultures or crude organelle preparations were placed on agarose cushions and visualized by phase contrast and epifluorescence microscopy using a Zeiss Axiovert 200 microscope. Note that for the analysis of organelle preparations, the agarose cushions were based on lysis buffer. Images were taken using a CCD camera (Hamamatsu Orca-ER) with an exposure time of 30–500 ms. Image acquisition was performed using Improvision Volocity software and processing was carried out with ImageJ. The quantification of co-localization of GFP signals and mCherry signals in organelle preparations was performed by manual inspection of mCherry-positive foci.

### Computational Analysis

Peroxisomal targeting signals 2 motifs were searched with the regular expression [RK][LVI].[HQ][LA]. Data analysis was performed with the National Center for Biotechnology Information (NCBI), basic local alignment search tool (Blast) (Altschul et al., 1990; Altschul and Koonin, 1998), and other NCBI resources. The RNA-Seq data of *U. maydis* FB1 and FB2 strains from axenic culture (GSM2785393) were retrieved from the Sequence Read Archive (NCBI). The data were first converted to the FASTA format and then searched for reads containing 20-mers mapping to the 300-bp 5′ UTR region of *pgd1* using Python 3.7.3 with Biopython 1.73 (Cock et al., 2009). These sequences were manipulated with notepad++ v7.8.4 using regular expression to identify unique reads. Reads containing an in-frame ATG start codon upstream of the dinucleotide AGG ATT in the pgd1 5′ UTR were filtered with the regular expression “ATG(.)^∗^AGGATT.” Box plots were computed using RStudio 3.6.0 with the ggplot2 plugin. The box plots are structured as follows: center line, median; box limits, first and third quartiles (the 25th and 75th percentiles); whiskers, 1.5^∗^ interquartile-range; points, all data points.

## Data Availability Statement

The datasets generated for this study are available on request to the corresponding author.

## Author Contributions

MK, EB, DM, TS, and JF performed the experiments and analyzed the data. AK contributed to bioinformatic analysis. MB and JF designed and supervised the study. MB and JF acquired funding. TS, MB, and JF wrote the manuscript.

## Conflict of Interest

The authors declare that the research was conducted in the absence of any commercial or financial relationships that could be construed as a potential conflict of interest.

## Supplementary Material

FIGURE S1 The PTS2 of Pgd1 is not activated by alternative splicing. **(A)** Published RNA-Seq data were filtered for reads containing 20-mers that mapped to the 300-bp fragment of the *pgd1* 5′ UTR (red). **(B)** Two out of 275 unique reads contain an in-frame ATG start codon (magenta) upstream of the PTS2 encoding sequence. The start codons are located within sequences derived from totally unrelated genes, suggesting artifactual reads (lowercase).

FIGURE S2 The mobility of GFP fusion proteins does not always reflect their molecular weight. **(A)** Fluorescence microscopic images of wild-type and Δ*pex7* cells expressing PTS2_*Pot2*_-GFP and mCherry-SKL. The scale bar represents 5 μm. **(B)** Whole-cell protein extracts from the indicated strains were analyzed by sodium dodecyl sulfate-polyacrylamide gel electrophoresis and Western Blot.

FIGURE S3 Subcellular localization of Pgd1-GFP and Sec63-GFP. **(A)** Cells co-expressing Pgd1-GFP under the control of the endogenous promoter and mCherry-SKL were analyzed by fluorescence microscopy. **(B)** Fluorescence microscopic images of cells expressing Sec63-GFP and the peroxisomal marker mCherry-SKL. The scale bars represents 5 μm.

TABLE S1 Strains used in this study.

TABLE S2 Oligonucleotides used in this study.
