# Supplementary figures and images for: Non-AUG Translation Initiation Generates Peroxisomal Isoforms of 6-Phosphogluconate Dehydrogenase in Fungi
Source: Front Cell Dev Biol. 2020 May 5;8:251. doi: 10.3389/fcell.2020.00251 (PMC7214817; doi:10.3389/fcell.2020.00251)

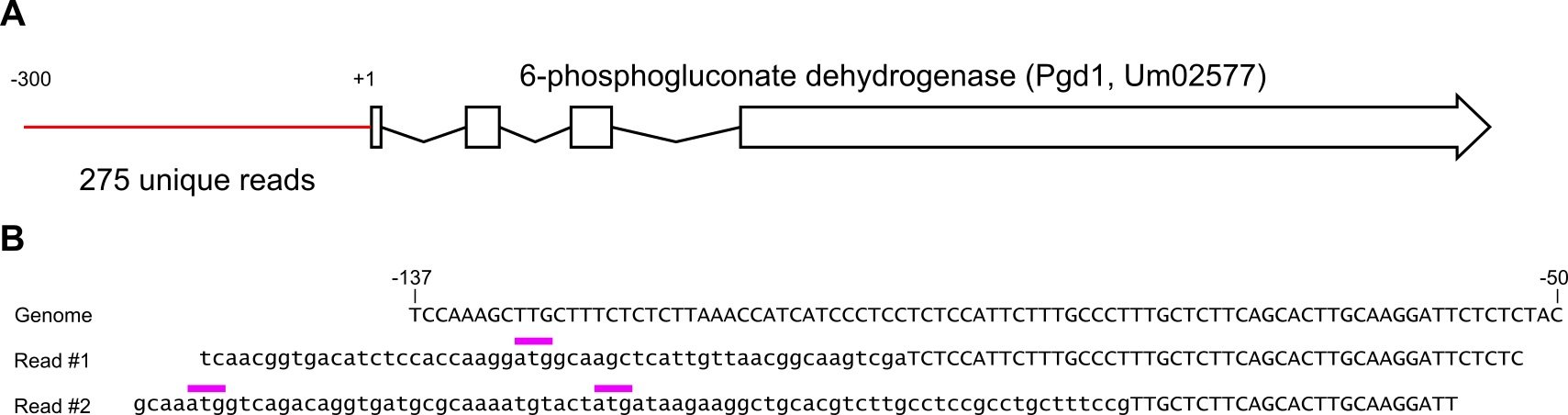

Supplement: FIGURE S1 — The PTS2 of Pgd1 is not activated by alternative splicing. (A) Published RNA-Seq data were filtered for reads containing 20-mers that mapped to the 300-bp fragment of the pgd1 5′ UTR (red). (B) Two out of 275 unique reads contain an in-frame ATG start codon (magenta) upstream of the PTS2 encoding sequence. The start codons are located within sequences derived from totally unrelated genes, suggesting artifactual reads (lowercase). [file Image_1.JPEG]

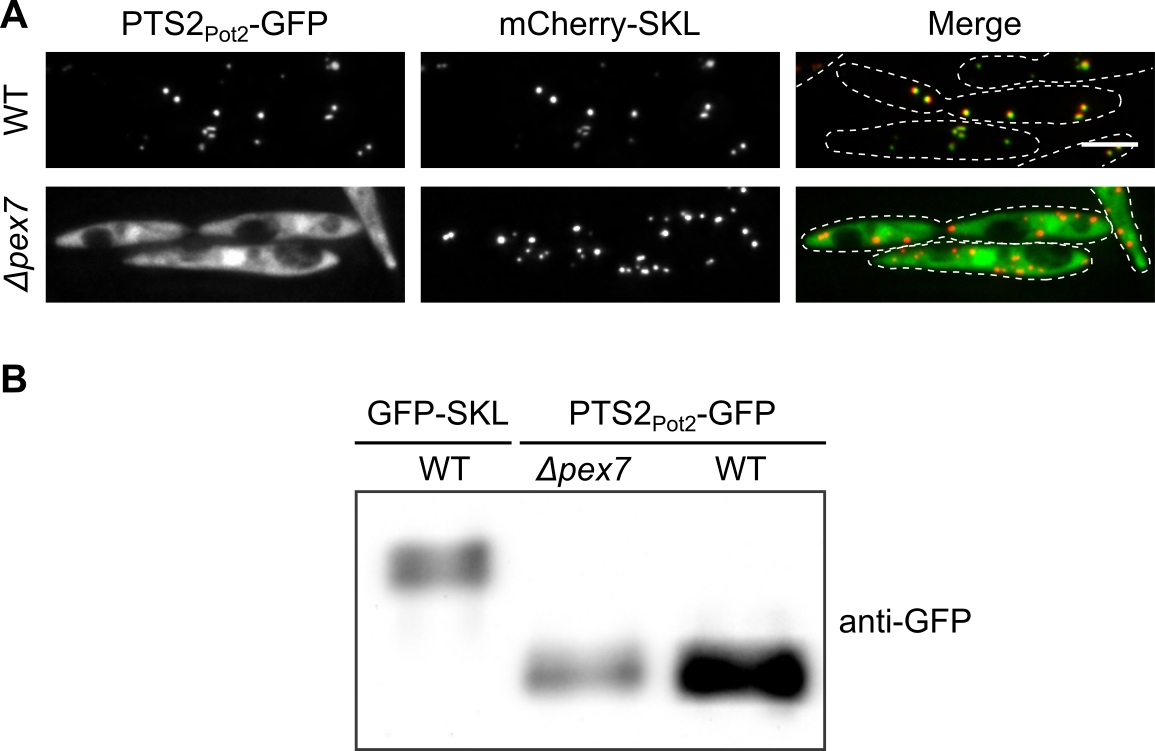

Supplement: FIGURE S2 — The mobility of GFP fusion proteins does not always reflect their molecular weight. (A) Fluorescence microscopic images of wild-type and Δpex7 cells expressing PTS2Pot2-GFP and mCherry-SKL. The scale bar represents 5 μm. (B) Whole-cell protein extracts from the indicated strains were analyzed by sodium dodecyl sulfate-polyacrylamide gel electrophoresis and Western Blot. [file Image_2.JPEG]

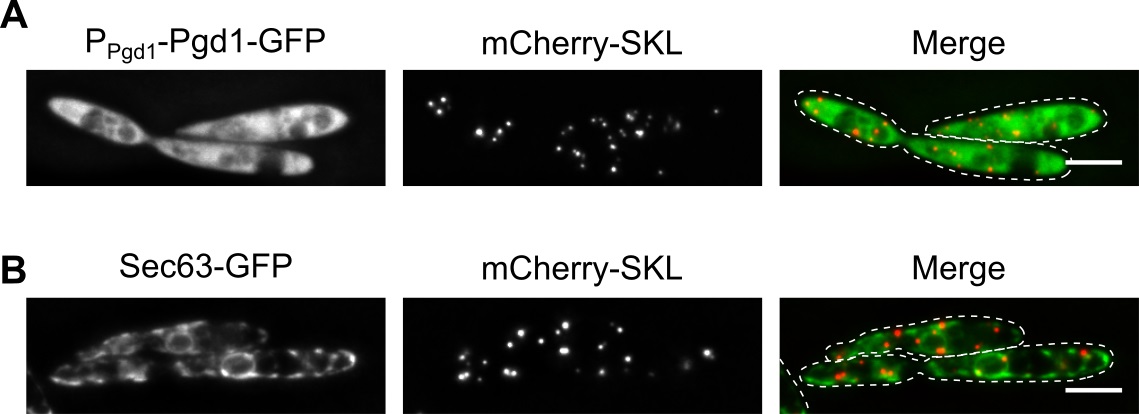

Supplement: FIGURE S3 — Subcellular localization of Pgd1-GFP and Sec63-GFP. (A) Cells co-expressing Pgd1-GFP under the control of the endogenous promoter and mCherry-SKL were analyzed by fluorescence microscopy. (B) Fluorescence microscopic images of cells expressing Sec63-GFP and the peroxisomal marker mCherry-SKL. The scale bars represents 5 μm. [file Image_3.JPEG]
